# High levels of sulphadoxine-pyrimethamine resistance *Pfdhfr-Pfdhps* quintuple mutations: a cross sectional survey of six regions in Tanzania

**DOI:** 10.1186/1475-2875-13-152

**Published:** 2014-04-21

**Authors:** Sungwa I Matondo, Godfrey S Temba, Adelaida A Kavishe, Julius S Kauki, Akili Kalinga, Marco van Zwetselaar, Hugh Reyburn, Reginald A Kavishe

**Affiliations:** 1 Kilimanjaro Christian Medical University College and Kilimanjaro Clinical Research Institute, Moshi, Tanzania; 2 Kilimanjaro Christian Medical Centre, Moshi, Tanzania; 3 National Institute for Medical Research, Tukuyu Centre, Tanzania; 4 London School of Hygiene and Tropical Medicine, London, UK

## Abstract

**Background:**

In 2006, the first-line anti-malarial drug treatment in Tanzania was changed from sulphadoxine-pyrimethamine (SP) to artemether-lumefantrine (ALu), an artemisinin-based combination (ACT), since when the use of SP has been restricted for intermittent preventive treatment in pregnancy (IPTp). A number of *Plasmodium falciparum* mutations are known to be associated with resistance to SP, but it is not known if the prevalence of these mutations is increasing or decreasing under the conditions of reduced levels of SP use. This study reports on the current SP resistant quintuple *Pfdhfr*-*Pfdhps* mutations in six regions of Tanzania.

**Methods:**

Finger-prick blood on filter paper and rapid diagnostic test strips from *P. falciparum*-positive individuals of all age groups attending health facilities in six regions of Tanzania between June 2010 and August 2011 were obtained. Using chelex-100 extracted DNA, genotyping was done for mutations on codons 51, 59 and 108 of *Pfdhfr* and 437 and 540 of *Pfdhps* genes using PCR-RFLP technique.

**Results:**

A total of 802 malaria-positive samples were screened and genotyped. The prevalence of *Pfdhfr* 51I, *Pfdhps* 437G and 540E varied between the regions (p < 0.001) whereas *Pfdhfr* 59R (FE 10.79, p = 0.225) and 108 N (FE 10.61, p = 0.239) did not vary between the regions. The *Pfdhfr* triple mutant was above 84% and close to fixation levels in all regions, whereas the *Pfdhps* double mutation ranged from 43.8 to 97% between the regions. The quintuple mutant (IRNGE) was the most prevalent in all regions and it varied significantly from 37.5 to 90.2% (*χ*^2^ = 1.11, p <0.001).

**Conclusions:**

There is evidence of persistent high levels of SP resistance markers in Tanzania with evidence of quintuple mutations that are likely to become fixed in the population. This threatens the future of SP not only in IPTp programmes, but as a combination drug for ACT. Continuous monitoring of SP-IPTp efficacy should be encouraged subsequent to searching for alternative drugs for IPTp in East Africa.

## Background

Tanzania introduced sulphadoxine-pyrimethamne (SP) as first-line treatment drug for uncomplicated malaria in 2001, replacing chloroquine (CQ), which had been the first-line since the 1970s [1]. Before it was declared first-line, SP was already in use as second-line drug and resistance had already developed [2,3]. This led to a rapid spread of SP resistance and eventually SP was replaced with the current artemisinin-based combinational therapy (ACT) by the end of 2006 [4]. Due to safety concerns for ACT use during pregnancy, especially in the first trimester, SP has continued to be used in intermittent preventive treatment of malaria in pregnancy (IPTp) and infants (IPTi). For IPTp, two or more doses of SP are administered after the first trimester at intervals of at least one month apart. The importance of SP-IPTp in prevention of malaria in pregnancy and the resulting outcomes, such as low birth weight, abortion, premature birth, perinatal death, and maternal mortality, have been documented globally and WHO has continued to recommend SP-IPTp use [5-8]. SP resistance has however continued to rise and several studies have reported reduced protection of SP-IPT programmes in areas where SP resistance is high [9-11].

SP resistance is caused by mutation on two genes, the dihydrofolate reductase (*Pfdhfr*) and the dihydropteroate synthetase (*Pfdhps*) genes. Three *Pfdhfr* mutations: N51I, C59R and S108N, referred to as the triple mutation, and the *Pfdhps* mutations: A437G and G540E, referred to as the double mutation, collectively form the quintuple mutations [12,13]. An additional mutation on *Pfdhps* 581 has been associated with high level of SP resistance and a strong predictor of SP-IPTp failure [14] and in addition to the quintuple forms the sextuple mutation. In East Africa SP resistance has reached over 90% and in some places the prevalence of the quintuple mutation is approaching fixation levels [15]. In Tanzania only two studies in Igombe-Mwanza and Korogwe-Tanga have documented the prevalence of quintuple mutation in 2008/2011 period. All other studies have used samples collected before or during the transition from SP to ACT in 2006. It is thus not clear whether SP resistance is decreasing or increasing in the advent of its restricted use. The current study set out to investigate the current SP resistance based on quintuple mutations in Tanzania.

## Methods

Samples collected through collaboration with ongoing studies in six regions of mainland Tanzania between June 2010 and August 2011 were used in this study. In Coastal Region the sample involved pregnant women attending the Kibiti health centre for intermittent preventive treatment of malaria. Sampling from all other regions involved all age groups. Finger-prick blood on filter paper (Whatman-3) or rapid diagnostic test kits (Mwanza samples) from febrile patients attending various health facilities in the respective regions were collected after patients’ or children’s guardians had consented to the use of their blood samples for malarial genetic studies. The study sites included Mwanza (Misungwi district) and Kagera (Muleba district) around Lake Victoria in the north-western zone, Tanga (Bondo village) in the north-eastern zone, Mtwara (Tandahimba and Mtwara-Urban) and Coastal Region (Kibiti-Rufiji) in the south-eastern zone, and Mbeya (Kyela and Rungwe districts) in the south-western zone. The malaria-positive rapid diagnostic test (RDT) strips or dried filter-paper blood spots were stored in desiccant at room temperature. Malaria parasite DNA was extracted using chelex-100 method as described previously [16]. Genotyping for *Pfdhps* and *Pfdhfr* was performed using PCR-RFLP methods described by others [17,18]. In short, nested PCR were performed followed by restriction digestion of the secondary products. For *Pfdhfr Tsp509I*, *XmnI* and *AluI* were used for positions 51, 59 and 108 respectively whereas for *Pfdhps* 437 and 540 *AvaII* and *FokI* were used, respectively. For each enzyme there were digestion control sites as previously described [17] in addition positive controls were used in each experiment. Digestion products were eluted on 2% agarose gel (Invitrogen, USA) stained with ethidium bromide and visualized under UV light. All PCR reagents and restriction endonucleases were purchased from New England Biolabs (Ipswich, MA, USA). Primers were purchased from Biolegio (Nijmegen, the Netherlands). Prevalence was calculated as the percentage of wild type or mutants out of the new total samples genotyped. Very few mixed infections were observed in this study and were excluded from the analysis as it was not possible to include them in haplotype analysis. The study received ethical approval from the Kilimanjaro Christian Medical University College Ethical Board subsequent to the National Institute for Medical Research Ethics approval obtained in the collaborating projects.

## Results

A total of 802 *P. falciparum* positive blood samples were screened and genotyped; 785, 787, 765, 762 and 752 were successfully genotyped for mutations at codons 51, 59, 108, 437 and 540 respectively; 707 (88%) of the 802 were successfully analyzed for the quintuple haplotypes. At codons 51, 59, 108 and 437, 0.6, 1.4, 1.3 and 1.4% of the genotyped samples had mixed genotypes. No mixed genotypes were observed at codon 540. Since the percentages were low, samples with mixed genotypes were excluded from haplotype calculation. Significant differences in prevalence of *Pfdhfr* 51I (FE 10.79, p < 0.001), *Pfdhps* 437G (*χ*^2^ = 1.5, p < 0.001) and 540E (*χ*^2^ = 1.12, p < 0.001) were observed between the regions. However, the prevalence of *Pfdhfr* 59R and 108 N mutations was not different between the regions (FE 10.79, p = 0.225 and FE 10.61, p = 0.239, respectively).

**Figure 1:**
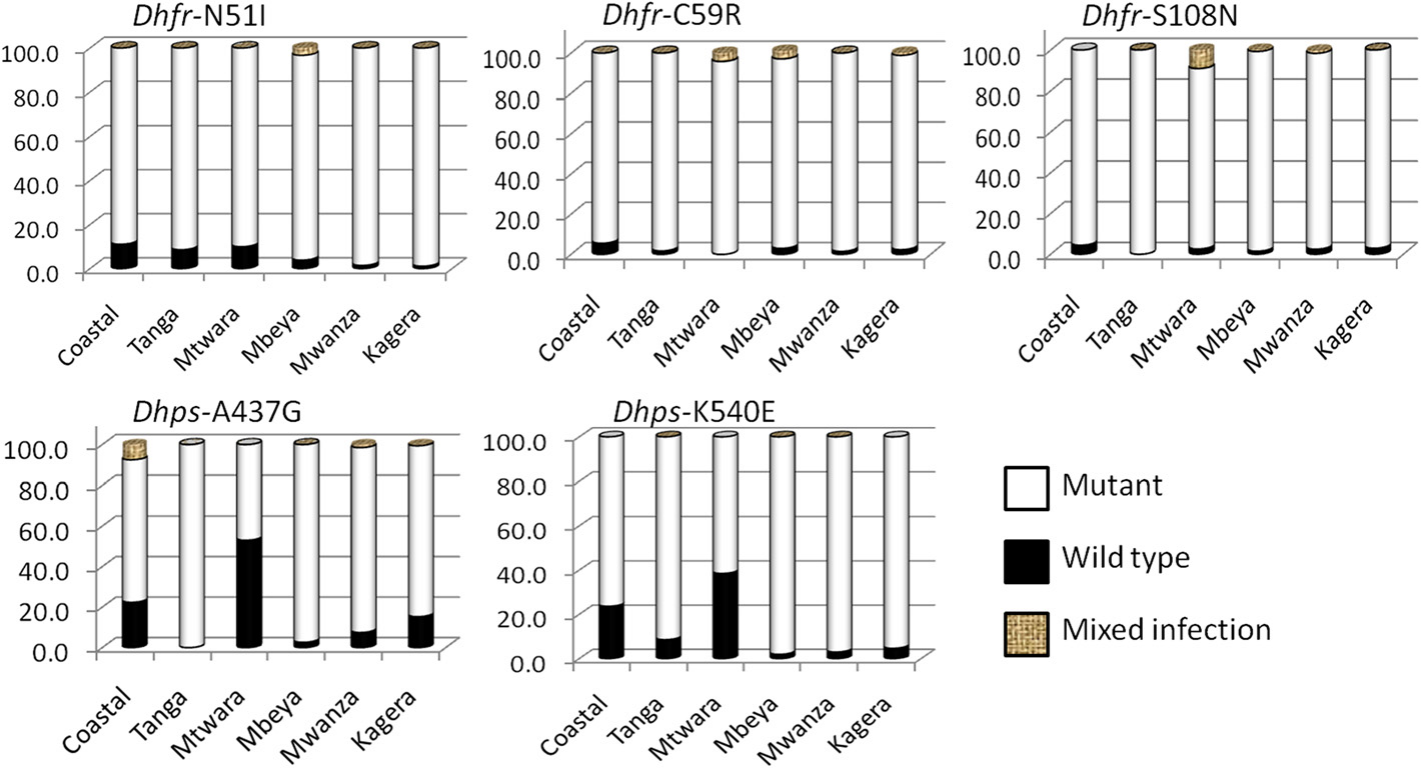
**Prevalence of *Pfdhfr *and *Pfdhps *mutations in Tanzania.** X-axis represents the six regions sampled and y-axis presents percentage prevalence calculated as total number of mutants or wild types per total number of samples per region.

*Pfdhfr* mutations were the most prevalent (Figure 1) with the triple mutant (IRN) ranging from 84.4 (Coastal) to 96.6% (Tanga) compared to *Pfdhps* double mutant (GE) which ranged from 43.8 to 97% (Table 1). Both the triple mutant and the double mutants were statistically different but when Coastal region was excluded the distribution of the IRN triple mutant was no longer different (FE 2.75, p = 0.594). The wild type *Pfdhfr* (NCS) and *Pfdhps* (AK) were detected at very low levels (0.1% and 5.1% respectively) (Table 1).

**Table 1:** **Prevalence of *Pfdhfr *triple and *Pfdhps *double mutants in Tanzania**

	***Pfdhfr *n (%)**							***Pfdhps *n (%)**				
**Regions**	**IRN**	**IRS**	**ICN**	**NRN**	**NCN**	**NCS**	**Total**	**GE**	**GK**	**AE**	**AK**	**Total**
Coastal	81 (84.4)	5 (5.2)	0 (0)	3 (3.1)	7 (7.3)	0 (0)	96	59 (61.5)	13 (13.5)	15 (15.6)	9 (9.4)	96
Tanga	112 (96.6)	0 (0)	2 (1.7)	2 (1.7)	0 (0)	0 (0)	116	107 (92.2)	9 (7.8)	0 (0)	0 (0)	116
Mtwara	59 (92.2)	2 (3.1)	0 (0)	3 (4.7)	0 (0)	0 (0)	64	28 (43.8)	8 (12.5)	12 (18.8)	16 (25.0)	64
Mbeya	127 (96.2)	3 (2.3)	2 (1.5)	0 (0)	0 (0)	0 (0)	132	128 (97.0)	1 (0.8)	3 (2.3)	0 (0)	132
Mwanza	126 (96.2	2 (1.5)	2 (1.5)	0 (0)	0 (0)	1 (0.8)	131	122 (93.1)	0 (0)	5 (3.8)	4 (3.1)	131
Kagera	158 (94.0)	6 (3.6)	4 (2.4)	0 (0)	0 (0)	0 (0)	168	148 (88.1)	1 (0.6)	12 (7.1)	7 (4.2)	168
Total	663 (93.8)	18 (2.5)	10 (1.4)	8 (1.1)	7 (1.0)	1 (0.1)	707 (100)	592 (83.7)	32 (4.5	47 (6.6)	36 (5.1)	707 (100)

**Figure 2:**
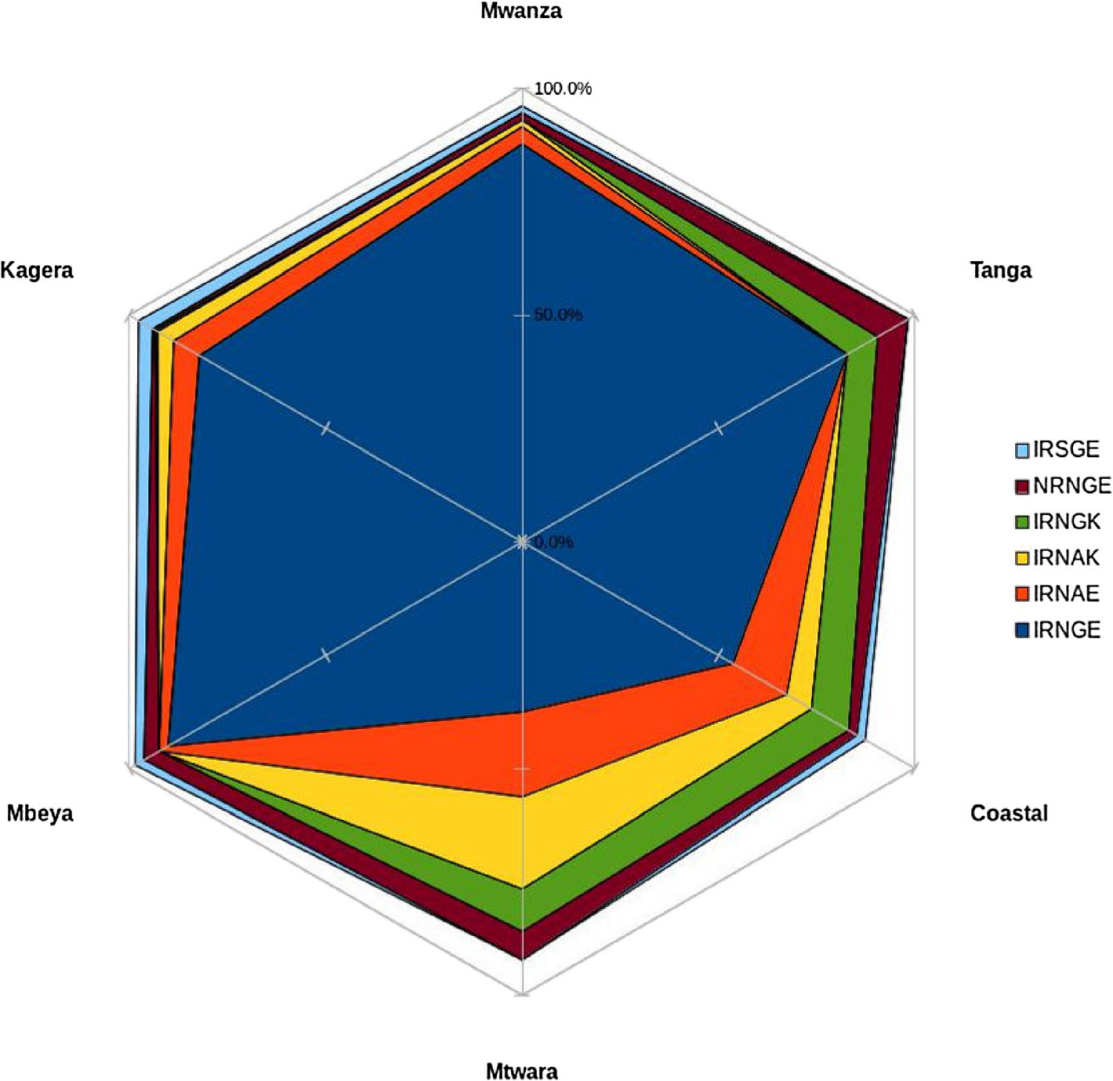
**Prevalence of *Pfdhfr*-*dhps *** **common quintuple haplotypes in Tanzania.**

Six common quintuple haplotypes were observed from the analysis (Table 2) with overall prevalence ranging from 1.8 to 76.9% depicted in Figure 2. An additional 13 minor haplotypes with prevalence less than 1% were grouped as “others” and constituted only 4.1% of the overall haplotypes. These include NRNGK (0.6%), IRSAK (0.4%), NCNGE (0.4%), NCNAK(0.3%), NCNGK (0.3%), NRNAE (0.1%), IRSAE (0.1%), IRSGK (0.1%), ICNGE (1.1%), NRNAK (0.1%), ICNGK (0.1%), NCSGE (0.1%) and ICNAE (0.1%). The IRNGE haplotype (quintuple mutant) was the most prevalent haplotype in all regions and it varied significantly across the regions (*χ*^2^ = 1.11, p < 0.001) (Table 2). Tanga, Mbeya, Mwanza and Kagera regions had the highest prevalence of the quintuple mutation compared to Coastal and Mtwara regions (Table 2 and Figure 2).

**Table 2:** **Prevalence of *Pfdhfr*-*Pfdhps *common haplotypes in six regions of Tanzania**

		**Common quintuple haplotypes n (%)**							**Total (N)**
		**IRNGE**	**NRNGE**	**IRNGK**	**IRSGE**	**IRNAE**	**IRNAK**	OTHER*	
**Regions**	Coastal	51 (53.7)	2 (2.1)	9 (9.5)	2 (2.1)	13 (13.7)	6 (6.3)	12 (12.6)	95
	Tanga	96 (82.8)	9 (7.8)	9 (7.8)	0 (0.0)	0 (0.0)	0 (0.0)	2 (1.7)	116
	Mtwara	24 (37.5)	4 (6.2)	6 (9.4)	0 (0.0)	12 (18.8)	13 (20.3)	5 (7.8)	64
	Mbeya	119 (90.2)	5 (3.8)	0 (0.0)	3 (2.3)	3 (2.3)	0 (0.0)	2 (1.5)	132
	Mwanza	115 (87.8)	2 (1.5)	0 (0.0)	2 (1.5)	5 (3.8)	2 (1.5)	5 (3.8)	131
	Kagera	138 (82.1)	1 (0.6)	1 (0.6)	6 (3.6)	11 (6.5)	7 (4.2)	4 (2.4)	168
Total		543 (76.9)	23 (3.3)	25 (3.5)	13 (1.8)	44 (6.2)	29 (4.1)	29 (4.1)	707

*Other haplotypes include: NRNGK, IRSAK, NCNGE, NCNAK, NCNGK, NRNAE, IRSAE, IRSGK, ICNGE, NRNAK, ICNGK, NCSGE and ICNAE.

## Discussion

Selection for SP resistance markers in Tanzania has remained high even after the replacement of SP for first-line treatment of uncomplicated malaria in 2006. The selection for individual *Pfdhfr* and *Pfdhps* mutations is very high throughout Tanzania. Comparing individual mutations, *Pfdhfr* 59R is already fixed in Mtwara region while 108 N and *Pfdhps* 437 are fixed in Tanga (Bondo). In Korogwe-Tanga, the 51I, 59R and 108 N were already above 95% in 2006 [14] and in Mbeya-Matema, in 2005 the 51I, 59R, 108 N, 437G, and 540E were 93, 80, 97.7, 78.6 and 77.4%, respectively [19]. A similar increase was observed in Mwanza Region. Between 2010 and 2011 the prevalence of 51I, 59R, 108 N, 437G, and 540E in Igombe-Mwanza was 75, 82.5, 94.8, 74, and 69.5%, respectively which is comparable to the current findings [20].

The wild type *Pfdhfr* haplotype NCS was reported at 1.9% in Tanga-Korogwe in the period 2008/2010 [21] but in this study it was not detected, it was detected in Mwanza at 0.8%. This indicates disappearance of the wild type haplotypes as the mutants increase.

Furthermore, compared to studies conducted between 2006 and 2007 around the time when SP was withdrawn as first line drug, the triple mutant (IRN) was 90 – 96.4% in Tanga (Korogwe), 74% in Coastal (Rufiji) and Mtwara/Lindi regions while in Mbeya (Matema) it was 82.6% in 2005 [19,22-24], thus there has been a continuous selection for the *Pfdhfr* triple mutants to date. Similarly, from around 2006 the double mutant (GE) and the quintuple respectively have continued to increase from 63 and 75% in Tanga [14,22], and 81 and 64% in Mbeya [19] while the GE increased from 57% in Lindi/Mtwara. There was no statistical difference in the distribution of the IRN across regions indicating homogeneity in SP selection pressure throughout the country.

The *Pfdhps* double (GE) mutant varied between the regions. While the prevalence was lower in Mtwara and Coastal regions, highest levels were observed in Mbeya, Mwanza, Tanga and Kagera. This may be accounted for by inter regional variations in the use of SP especially during or before SP became first line treatment drug. Before 2001 SP was second line drug and CQ was the first line. During this time SP resistance had already occurred. This contributed to a rapid spread of resistance after SP was made first line in 2001. In 2005 Mbeya registered exceptionally high levels of GE (81%) [19] and in the current study Mbeya is the leading with highest levels of SP resistance (Tables 1 and 2, Figure 1).

Six common quintuple haplotypes were observed. The observed high levels of the quintuple mutation in all regions derive from the high levels observed with the triple and double mutations of *Pfdhfr* and *Pfdhps*. 7The low levels of double mutant (GE) in Coastal and Mtwara regions resulted into low levels of the quintuple mutation in these regions. These findings are comparable to recent studies in other East African countries. In western Kenya samples obtained from pregnant women between 2008 and 2009 were found to harbour more than 90% *Pfdhps* double mutant and more than 80% quintuple mutation [25]. In Mozambique SP resistance quintuple mutation was reported to be above 75% in 2008 while the triple mutation had reached 100% (fixation) [26]. These reports point to high SP resistance in the East African region as opposed to the West African region where SP resistance based on the quintuple mutation is still low in most countries, thus SP-IPT is still effective [27-29].

The prevalence of the quintuple mutation in the parasite confers high level SP resistance. In East Africa high levels of this haplotype are likely to compromise the importance of SP-IPTp [30]. Several studies have shown that although implementation of SP-IPTp does not prevent malaria infection during pregnancy, especially in the presence of high prevalence of SP-resistance markers [14,31,32], there is a significant protection against severe outcomes of pregnancy in malaria, such as low birth weight, maternal and neonatal mortality, especially when more than two doses of IPTp are administered [33]. This led to WHO’s continued recommendation for SP-IPTp at any level of quintuple mutations [34]. However, continued SP-IPTp is likely to exacerbate the spread of the highly resistant *Pfdhps* mutation 581 previously reported to associate with IPTp failure in East Africa [14,25]. Thus, aside from the WHO recommended > two doses of SP-IPTp, the high prevalence of SP resistance markers observed in Tanzania and elsewhere in East Africa calls for careful and continuous evaluation of SP-IPTp efficacy and on the usefulness of SP in artemisinin combinations. There is a need to screen pregnant mothers for malaria parasites even when they are already on IPTp in order to identify early treatment failure of the intervention [35]. Recent studies show that CQ withdrawal from use for a number of years has reversed resistance based on prevalence of *Pfcrt* resistance marker [36,37]. This was possible since CQ use was totally banned making its availability to both health facilities and local drug vendors difficult. A survey done in 2007 documented CQ use in Tanzania at 0.5% and in Malawi at 0.8% [38]. This led to the reported recovery of CQ susceptibility in Tanzania and Malawi. Conversely, due to continued use of SP for IPTp, SP is readily available in both public and the private sector making its restriction to only IPTp impossible. In the current situation it is unlikely that self-medication with SP can be prevented especially due to its low cost compared to ACT, which may also explain the observed high prevalence of SP resistance markers despite its replacement with ACT. Use of SP-artesunate combination is also another selection factor for SP-resistance markers, however, in Tanzania SP-AS is not used instead artemether-lumefantrine (ALu) is the approved ACT. Furthermore, it is expected as the quintuple mutation continues to rise towards fixation, the *Pfdhps* 581G mutation considered to confer SP super-resistance when in combination with the 540E will continue to rise. It is important for the responsible authorities to consider restricting SP to IPTp only, through restricting its general prescription and its availability to local drug vendors. An alternative drug for IPTp is urgently needed.

## Conclusion

In this study prevalence of SP resistance based on quintuple mutations in Tanzania is high, approaching fixation levels. This trend has been observed in other parts of East Africa. The spread of SP super-resistance is expected with continued SP use and may lead to poor SP-IPTp outcome despite continued recommendation by the WHO. An urgent search for alternative drugs for IPTp in East Africa is required.

## Competing interests

The authors have declared that they have no competing interests.

## Authors’ contributions

SIM participated in study design, performed the experiments, interpreted the data and drafted the manuscript. GST participated in performing the experiments and revised the manuscript. AAK and AK supervised sample collection in the field and revised the manuscript. JSK and MvS participated in data analysis and reviewed the manuscript. HR participated in study design and reviewed the manuscript. RAK conceived the idea, designed the study, analysed the data and wrote the manuscript. All authors read and approved the final version of the manuscript.
